# Leaf Health Status Regulates Endophytic Microbial Community Structure, Network Complexity, and Assembly Processes in the Leaves of the Rare and Endangered Plant Species *Abies fanjingshanensis*

**DOI:** 10.3390/microorganisms12071254

**Published:** 2024-06-21

**Authors:** Long Li, Rong Zheng, Zuhua Wang, Haibo Li, Yongjia Shi, Zhongjie Pan, Min Liu

**Affiliations:** 1 School of Data Science, Tongren University, Tongren 554300, China; lg874525275@163.com; 2 College of Life Science and Technology, Inner Mongolia Normal University, Hohhot 010022, China; zhengrong09@163.com; 3 College of A&F Engineering and Planning, Tongren University, Tongren 554300, China; zuhua131666@126.com (Z.W.); syj010203@foxmail.com (Y.S.); panzj0301@163.com (Z.P.); 4 National Nature Reserve Administration of Fanjing Mountain, Tongren 554400, China; fjsgljlhb@126.com

**Keywords:** *Abies fanjingshanensis*, leaf health status, leaf endophytic microbiomes, co-occurrence network, community assembly

## Abstract

The rare and endangered plant species *Abies fanjingshanensis*, which has a limited habitat, a limited distribution area, and a small population, is under severe threat, particularly due to poor leaf health. The plant endophytic microbiome is an integral part of the host, and increasing evidence indicates that the interplay between plants and endophytic microbes is a key determinant for sustaining plant fitness. However, little attention has been given to the differences in the endophytic microbial community structure, network complexity, and assembly processes in leaves with different leaf health statuses. Here, we investigated the endophytic bacterial and fungal communities in healthy leaves (HLs) and non-healthy leaves (NLs) of *A. fanjingshanensis* using 16S rRNA gene and internal transcribed spacer sequencing and evaluated how leaf health status affects the co-occurrence patterns and assembly processes of leaf endophytic microbial communities based on the co-occurrence networks, the niche breadth index, a neutral community model, and C-score metrics. HLs had significantly greater endophytic bacterial and fungal abundance and diversity than NLs, and there were significant differences in the endophytic microbial communities between HLs and NLs. Leaf-health-sensitive endophytic microbes were taxonomically diverse and were mainly grouped into four ecological clusters according to leaf health status. Poor leaf health reduced the complexity of the endophytic bacterial and fungal community networks, as reflected by a decrease in network nodes and edges and an increase in degrees of betweenness and assortativity. The stochastic processes of endophytic bacterial and fungal community assembly were weakened, and the deterministic processes became more important with declining leaf health. These results have important implications for understanding the ecological patterns and interactions of endophytic microbial communities in response to changing leaf health status and provide opportunities for further studies on exploiting plant endophytic microbes to conserve this endangered *Abies* species.

## 1. Introduction

Endophytic microbes are functionally dominant members of the plant microbiome that colonize internal plant tissues for all or at least a part of their life cycle without causing any symptoms of disease in the host [1]. Plant endophytes are essential for plant growth and development, plant community biodiversity, and ecosystem functions [2,3,4]. Leaf endophytic bacteria and fungi are major components of the diverse plant endophyte communities that have been found in all plant species sampled to date [5]. They contribute to plant growth and resistance by modifying defensive features against biotic and abiotic stresses, regulating leaf photosynthetic rates, and affecting nutrient uptake and distribution within host tissues [2,5,6,7]. Differences in leaf endophytic microbial communities are strongly influenced by plant species, leaf traits, plant secondary metabolites, and abiotic environmental variables [8,9,10,11,12]. Furthermore, recent studies have suggested that the diversity and compositional structure of the leaf endophytic microbiome show considerable variations with changes in leaf health status. Yang et al. [13] showed that bacterial blight induced shifts in endophytic fungi, with saprophytic fungi (e.g., *Khuskia* spp. and *Leptosphaerulina* spp.) being enriched in bacterial blight-diseased rice leaves, whereas plant pathogens or mycotoxin-producing fungi (e.g., *Fusarium*, *Magnaporthe*, and *Aspergillus*) were found to be significantly more abundant in healthy rice leaves. Humphrey and Whiteman [14] indicated that native bittercress damaged by *Scaptomyza nigrita* had an overall greater bacterial density than undamaged leaves, mainly due to the increased abundance of typical leaf bacteria, particularly *Pseudomonas*. As the interactions between the endophytic microbiome and the leaves of host plants represent important aspects of plant fitness maintenance, it is important to elucidate the mechanisms that shape the composition of endophytic microbial communities in leaves of different health status to understand their ecological functions in mitigating leaf decline.

In general, highly complex microbial networks have the ability to resist biotic and abiotic perturbations to hosts and maintain plant health, and previous studies have shown that more complex and highly connected co-occurrence networks are consistently observed in healthy plant tissues and in soils [15,16]. In addition, a recent plastic greenhouse study showed that the network characteristics of the bacterial and fungal communities were differentially affected by plant health status in the soils of lisianthus with different disease incidence plots [17]. However, with regard to leaf endophytic microbes, recent studies have been focused mainly on the effects of the endophytic bacterial communities or fungal communities on plant leaf health [18,19], and the cooperative interactions of endophytic bacterial and fungal communities in response to changes in leaf health have been overlooked. Furthermore, evidence has shown that microbial taxa are sensitive to specific plant factors (e.g., plant compartment niches and plant health status), which may be considered of ecological importance in microbe–microbe association shifts, and some of these sensitive microbial taxa play important roles in determining basic host functions [20,21]. Therefore, it is necessary to identify endophytic microbiota that are sensitive to changes in plant health to provide an approach to obtain beneficial bacteria and fungi for maintaining leaf health while circumventing large-scale microbial cultivation.

Microbial community assembly is simultaneously shaped by both deterministic and stochastic processes. Deterministic processes are niche-based and determine community assembly through environmental filtering and interspecies interactions, whereas stochastic processes highlight that microbial communities are governed by the largely random processes of birth–death events, ecological drift, and probabilistic dispersal according to neutral theory [22,23]. A large body of literature has demonstrated the assembly processes of belowground soil and root-associated microbial communities in a variety of terrestrial ecosystems [24,25,26,27,28], but the relative importance of deterministic and stochastic processes in microbial community assembly may vary in different ecological niches, i.e., from the bulk and rhizosphere soils to the roots and leaves of host plants [26,29]. For plant endophytic microbial communities, the microenvironment of different plant tissues appears to be one of the most important factors responsible for determining which microbes can colonize internal tissues [30]. However, the relative importance of stochastic and deterministic processes in how the assembly of leaf endophytic microbial communities shifts with changes in leaf health status remains unclear.

*A. fanjingshanensis* is listed as in danger of extinction by the IUCN Red List of Threatened Species, and it is also a Grade 1 protected plant in China, which is found only near the summit area of the Fanjingshan National Nature Reserve in Southwest China. As an ancient Tertiary relict plant, *A. fanjingshanensis* has been important in tracing the origin and evolutionary history of rare and endangered *Abies* species in subtropical regions [31]. However, the population size of this species has decreased since the discovery of *A. fanjingshanensis*, possibly due to global climate change, harsh environmental conditions with poor soils and low temperatures, and poor community stability [32,33,34]. Although the exact mechanisms underlying its decline are largely unknown, many studies have confirmed that the health status of this tree species is under severe threat, especially that of its needles [33,34,35]. Previously, researchers have investigated differences in photosynthesis, nutrient characteristics, and the primary and secondary metabolites found in leaves with different leaf health statuses in crops or fruit trees [19,36,37,38], but there are few studies on the impact of leaf health status variation on endophytic microbial communities in coniferous tree species. Accordingly, both healthy and non-healthy leaves of *A. fanjingshanensis* were collected to analyze the leaf endophytic microbial communities using an Illumina NovaSeq platform, and the objectives of the current study were to answer the following questions: (a) Does leaf health status have a significant effect on the diversity and structure of endophytic microbial communities? (b) Which endophytic microbial taxa are sensitive to changes in leaf health? (c) How does leaf health status affect the complexity of co-occurrence networks and the relative importance of deterministic and stochastic processes in governing leaf endophytic microbial community assembly?

## 2. Materials and Methods

### 2.1. Study Site and Sample Collection

Fanjing Mountain (27.78~28.02° N, 108.60~108.81° E) is located within the Wuling mountain range in the northeastern part of Guizhou Province (Southwest China), and is a national nature reserve and a world natural heritage property. The elevation at Fanjing Mountain ranges from 500 m to 2570 m above sea level, and thus, the vegetation is vertically stratified into five typical zones: evergreen broadleaf forest, mixed evergreen and deciduous broadleaf forest, deciduous broadleaf forest, subalpine coniferous forest, and alpine shrub meadow. Fanjing Mountain contains the richest biodiversity in the world at the same latitude, with a total of 4394 plant and 2767 animal species recorded. It also has one of the highest concentrations of gymnosperms in the world and is the only ‘refuge’ for *A. fanjingshanensis*. The sampling area was located on Lanchaling, where *A. fanjingshanensis* is concentrated. The region is characterized by cool and humid temperatures and co-occurring rain and heat, with an average annual temperature of 7.07 °C, an average annual precipitation of 1313.8 mm, and an average annual relative humidity of 80.75%. The soils are mainly mountainous yellow-brown soil, with shallow and moist soil layers. The vegetation type of this region is temperate mixed coniferous and broad forest, and *A. fanjingshanensis*, *Tsuga chinensis*, *Enkianthus quinqueflorus,* and *Fargesia spathacea* are the dominant species.

Twenty trees were randomly selected for sampling from a 50 × 50 m plot on the shady slope of Lanchaling. The selection criteria were that the trees had similar heights and diameters at breast height, were healthy in appearance, and were in good physiological condition. To guarantee sample independence, we ensured that the distance between each tree was at least 10 m. Tree branches of similar ages containing both healthy and non-healthy leaves were randomly selected at the same tree height from each of the four directions. Healthy leaves (HLs) had a deep green color and no obvious symptoms of disease, whereas non-healthy leaves (NLs) had a yellow color and at least half the length of the needles were dead. For each branch, approximately 10 g of leaves of HL and NL was collected, and then the four replicate subsamples of leaves with the same health status were combined into one sample, resulting in a total of 40 leaf samples. The leaves were packed in sterilized bags, transported to the laboratory on ice within a few hours, and frozen at −80 °C for microbial DNA extraction.

### 2.2. DNA Extraction, PCR Amplification, Illumina Sequencing, and Data Processing

To remove surface microbial DNA, the leaf samples were first rinsed with sterile water and then placed in 70% (*v*/*v*) ethanol for 1 min, 3.3% sodium hypochlorite for 3 min, and 75% ethanol for 30 s [39]. The leaf samples were further washed three times in sterile distilled water for 2 min each time, and then the final rinse water was plated separately on potato dextrose agar and nutrient agar to evaluate the effectiveness of surface sterilization by the absence of microorganism growth on the culture media [39]. The successfully surface-sterilized leaves from the same sample were then homogenized using a Geno/Grinder 2010 (SPEX SamplePrep, Metuchen, NJ, USA) at 1200 strokes for 6 × 40 s. Total DNA was extracted from approximately 0.1 g of randomly selected homogenized leaves using a DNeasy^®^ Plant Mini Kit (Qiagen Gmbh, Hilden, Germany) according to the manufacturer’s instructions. The quality of the extracted DNA was measured by 1.0% agarose gel electrophoresis, and a NanoDrop 2000 spectrophotometer (Thermo Fisher Scientific, Waltham, MA, USA) was used to determine the purity and concentration of the DNA. The V3-V4 regions of the bacterial 16S rRNA gene and the internal transcribed spacer 2 region of the fungal small-subunit rRNA genes were amplified using the universal primers 341F (5′-CCTACGGGNGGCWGCAG-3′)/805R (5′-GACTACHVGGGTATCTAATCC-3′) and ITS1FI2 (5′-GTGARTCATCGAATCTTTG-3′)/ITS2 (5′-TCCTCCGCTTATTGATATATGC-3′), respectively. PCR reactions were carried out in 25 μL reaction mixtures containing 12.5 μL Phusion^®^ Hot Start Flex 2X Master Mix (New England Biolabs Inc., Ipswich, MA, USA), 50 ng extracted DNA template, 2.5 μL of each primer (10 μm), and distilled water to a final volume of 25 μL. The PCR conditions consisted of an initial denaturation temperature of 98 °C for 30 s followed by 35 cycles of 98 °C for 10 s, 54 °C for 30 s, 72 °C for 45 s, and a final 10 min extension step at 72 °C. The PCR products were separated by gel electrophoresis on a 2% agarose gel and purified using the AxyPrep DNA Gel Extraction Kit (Axygen Biosciences, Union City, CA, USA). The purified PCR products were quantified using QuantiFluor-ST (Promega, Fitchburg, WI, USA) and then subjected to library preparation. Library construction and paired-end sequencing (2 × 250 bp) on an Illumina NovaSeq 6000 platform were completed by LC-Bio Technology Co., Ltd. (Hangzhou, China).

Raw microbial sequences were processed using QIIME 2 (quantitative insights into microbial ecology) [40]. ITS2 region was extracted using the q2-ITSxpress plugin for the fungal data set [41]. DADA2 (divisive amplicon denoising algorithm) was used for denoise and chimera removal, and then the reads were clustered into amplicon sequence variants (ASVs) [42]. Feature sequences of ASVs and ASV tables were merged, and singleton ASVs were removed. Endophytic bacterial and fungal ASVs were taxonomically assigned at ≥99% similarity using a naïve Bayes classifier based on the SILVA and UNITE databases, respectively. Endophytic microbial ASVs that were not assigned to a phylum and were classified as chloroplasts or mitochondria were excluded from subsequent analysis.

### 2.3. Statistical Analysis

All statistical analyses were performed using various packages within the R (Version 4.3.2) statistical computing environment (http://www.r-project.org accessed on 30 January 2024). Differences in endophytic microbial alpha diversity between different leaf health statuses were analyzed by an independent samples *t*-test. Nonmetric multidimensional scaling (NMDS) and a permutational multivariate analysis of variance (PERMANOVA) with 999 permutations were performed based on the Bray–Curtis distance matrices using the vegan package (Version 2.6-4) [43] to evaluate the effect of health status on endophytic microbial community structures.

Prior to constructing the co-occurrence network, two complementary methods were employed to identify the endophytic microbial ASVs that were most responsive to health status. Indicator species analysis was performed in the indicspecies package (Version 1.7.14) [44] to detect indicator ASVs associated with leaves of different health status with 9999 permutations and differences considered significant at *p* < 0.05. Differential abundances of endophytic microbial ASVs were tested between leaves of different health status using the likelihood ratio test at a false discovery rate correction value of *p* < 0.05 in the edgeR package (Version 4.0.3) [45]. Thereafter, the ASVs confirmed by both analyses were defined as ‘health-sensitive ASVs (hsASVs)’. To elucidate the distribution patterns of hsASVs specific to leaves with different health statuses, a co-occurrence network including both bacterial and fungal communities was constructed following the approach of Hartmant et al. [46]. We utilized the trimmed means of M (TMM) normalized counts per million (CPM) counts in the edgeR package [45] and calculated Spearman’s rank correlations between all pairs of bacterial ASVs and all pairs of fungal ASVs using the Hmisc package (Version 5.1-1) [47]. Greedy optimization of the modularity algorithm was used to identify major network modules within the networks as implemented in the igraph package (Version 2.0.3) by the cluster_fast_greedy function [48]. Positive, significant correlations (r > 0.6 and *p* < 0.001) and major modules in the network were visualized using the Fruchterman–Reingold layout with 9999 permutations in the igraph package [48]. The co-occurrence networks of endophytic bacterial and fungal communities in HLs and NLs were subsequently constructed using the WGCNA package (Version 1.72-5) [49] based on Spearman’s correlation matrices according to the analytical methods reported by Qiu et al. [50]. Only ASVs present in at least 5 samples with at least 2 reads for bacteria and in at least 3 samples with at least 2 reads for fungi were included in the networks. Robust correlations with strong Spearman’s correlation coefficients (bacteria, r > 0.8; fungi, r > 0.7) and high statistical significance (*p* < 0.01) were used to generate the networks. The topological characteristics of the networks were obtained using the igraph package [48].

To reveal the distribution of ASVs in HLs and NLs, Levins’ niche breadth (*B*) index [51] within bacterial and fungal communities was calculated according to the following formula:

$$Bj=\frac{1}{\sum_{i=1}^{N}P_{ij}^{2}}$$

where *Bj* is the habitat niche breadth of ASV *j* in a meta-community, *N* is the total number of communities in each meta-community, and *P_ij_* is the proportion of ASV *j* in community *i*. A higher *B*-value of ASVs indicates that the ASV occurs widely and evenly across a wide range of habitats, representing a wide habitat niche breadth. The community niche breadth was calculated as the average of *B*-values of all the ASVs within a single community. This analysis was performed using the niche.width function within the spaa package (Version 0.2.2) [52].

A neutral community model (NCM) was used to estimate the potential contribution of stochastic processes in shaping the assembly of leaf endophytic microbial communities in HLs and NLs by predicting the relationships between the observed frequency of ASV occurrence in a set of local communities and their relative abundance across the wider metacommunity [53]. The 95% confidence intervals of all fitting statistics were calculated based on 1000 bootstrap replicates. NCM was performed in R using the code provided by Burns et al. [54]. To explore the clustering or overdispersion of endophytic bacterial and fungal communities in HLs and NLs, the checkerboard score (C-score) was calculated by examining the deviation of each observed metric from the average of this null model. The standardized effect size (SES) for the C-score was evaluated as the difference between the observed index and the mean of the stimulated index over the standard deviation of the stimulated index, and C-score and SES analyses were calculated based on a binary matrix of ASV presence (1) and absence (0). The C-score was based on 30,000 simulations with a burn-in of 500 iterations and evaluated using a sequential swap randomization algorithm with the EcoSimR package (Version 0.1.0) [55].

## 3. Results

### 3.1. Composition of the Leaf Endophytic Microbial Community

A total of 2,034,410 high-quality bacterial sequences (ranging from 8095 to 75,493 sequences per sample) and 780,988 high-quality fungal sequences (ranging from 5828 to 41,812 sequences per sample) were obtained, and they were separated into 3602 endophytic bacterial ASVs (ranging from 166 to 733 ASVs per sample) and 2279 endophytic fungal ASVs (ranging from 76 to 242 ASVs per sample) in all leaf samples. The rarefaction curves of most leaf samples appeared to reach saturation, indicating that we had obtained the majority of endophytic microbial ASVs (Figure S1).

**Figure 1:**
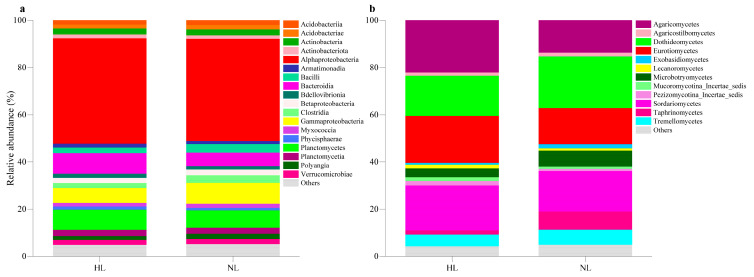
Relative abundance of leaf endophytic bacterial (**a**) and fungal (**b**) communities at the class level in healthy leaves (HLs) and non-healthy leaves (NLs).

The dominant phyla of endophytic bacterial communities were Proteobacteria (HL: 54.92%, NL: 56.54%) and Planctomycetota (HL: 12.74%, NL: 11.18%). Alphaproteobacteria (HL: 45.52%, NL: 44.12%) were the dominant class detected in HLs and NLs (Figure 1a). However, the effect of leaf health status on the relative abundance of Alphaproteobacteria was not significant (Wilcoxon rank sum tests, *p* > 0.05). Ascomycota were the most abundant endophytic fungal phylum, with 62.44% in HLs and 65.43% in NLs, followed by Basidiomycota (HL: 35.04%, NL: 33.07%), Zygomycota (HL: 1.64%, NL: 1.03%), and Chytridiomycota (HL: 0.88%, NL: 0.47%). Agaricomycetes (HL: 22.10%, NL: 13.61%), Eurotiomycetes (HL: 19.89%, NL: 15.35%), Sordariomycetes (HL: 19.00%, NL: 17.09%), and Dothideomycetes (HL: 16.92%, NL: 21.76%) were the dominant classes in both HLs and NLs (Figure 1b). Poor leaf health significantly increased the relative abundance of Dothideomycetes and decreased the relative abundance of Agaricomycetes and Eurotiomycetes (Wilcoxon rank-sum test, *p* < 0.001), but there was no significant difference in the relative abundance of Sordariomycetes between HLs and NLs (Wilcoxon rank-sum test, *p* > 0.05).

### 3.2. Diversity of the Leaf Endophytic Microbial Community

**Figure 2:**
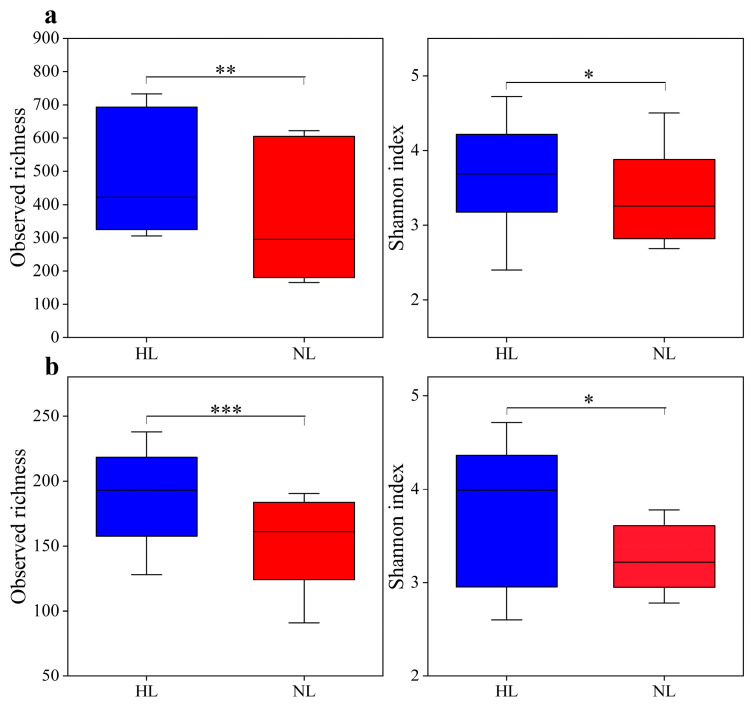
Leaf endophytic bacterial (**a**) and fungal (**b**) richness and Shannon index in healthy leaves (HLs) and non-healthy leaves (NLs). Significant differences were analyzed by independent samples *t*-test; *, **, and *** indicate *p* < 0.05, <0.01, and <0.001, respectively.

**Figure 3:**
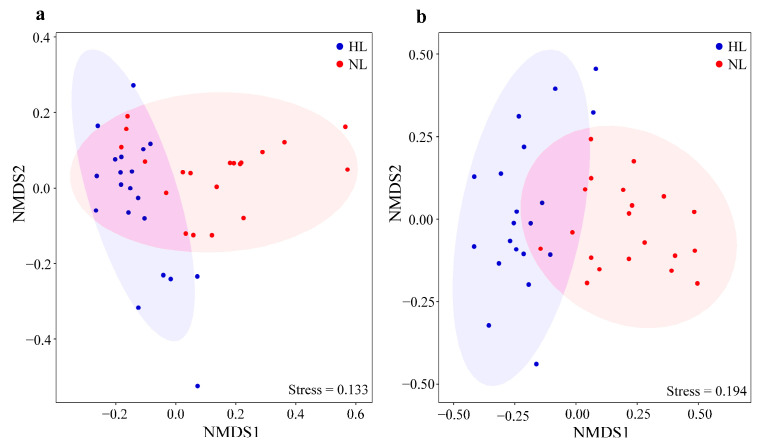
Leaf endophytic bacterial (**a**) and fungal (**b**) community structures based on the Bray–Curtis distance in healthy leaves (HLs) and non-healthy leaves (NLs). The ellipses represent 95% confidence intervals of the sample ordinations grouped by leaf health status.

The ASV richness and Shannon index of the endophytic bacterial and fungal communities showed a similar pattern with respect to leaf health status, and these indices were significantly higher in HLs than in NLs (Figure 2). NMDS analysis revealed that both the endophytic bacterial (Figure 3a) and fungal (Figure 3b) communities were clearly separated between HLs and NLs. This distribution pattern was supported by the PERMANOVA results (bacteria: R^2^ = 0.142, *F* = 6.307, *p* < 0.001; fungi: R^2^ = 0.239, *F* = 11.965, *p* < 0.001), indicating that health status shaped the leaf microbiome. The differences were greater for endophytic fungal communities than for endophytic bacterial communities, suggesting that endophytic fungal communities were more sensitive to leaf health status.

### 3.3. Co-Occurrence and Network Complexity of the Leaf Endophytic Microbial Community

**Figure 4:**
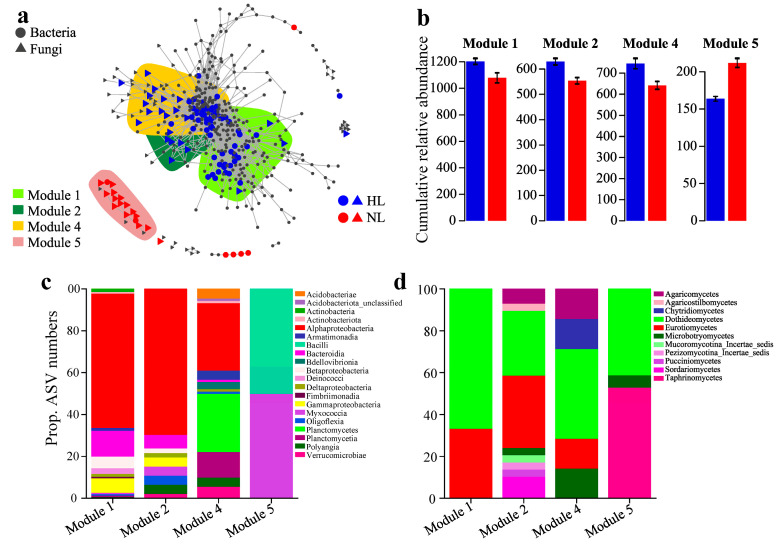
Co-occurrence patterns of the health-sensitive amplicon sequence variants (ASVs). (**a**) Visualization of sensitive ASVs in leaves of different health status using co-occurrence networks based on significant correlations (r > 0.6, *p* < 0.001; indicated by gray lines) between endophytic bacterial and fungal ASVs in leaves. Circles and triangles indicate endophytic bacterial and fungal ASVs, respectively. The sensitive endophytic bacterial and fungal ASVs that are abundant under specific leaf health status are colored blue for healthy leaves (HLs) and red for non-healthy leaves (NLs), and gray ASVs are those insensitive to health status. The shaded areas represent the network modules containing sensitive ASVs. (**b**) Overall responses of sensitive modules to different leaf health statuses are indicated by the cumulative relative abundance (as counts per million, CPM; *y*-axis in ×1000) of all endophytic bacteria and fungi of the health-sensitive modules. The error bars represent the SDs. (**c**,**d**) The qualitative taxonomic composition of the health-sensitive modules is given as the proportional ASV numbers per class of endophytic bacteria (**c**) and fungi (**d**).

The distribution patterns of the hsASVs (Tables S1 and S2) in the meta co-occurrence networks of the endophytic bacterial and fungal communities in the leaves were investigated (Figure 4 and Table S3). Four modules (modules 1, 2, 4, and 5) with relatively high proportions of hsASVs were identified in the meta-networks (Figure 4a, Figure S2). The relative abundance of these sensitive modules (Figure 4b) and their distributions in the network (Figure 4a) partially revealed that the dissimilarity of the endophytic microbial communities was mainly driven by leaf health status, consistent with the NMDS and PERMANOVA results. For example, numerous hsASVs assigned to HLs were mainly located in modules 1, 2, and 4, and were separated from module 5, which primarily contained ASVs sensitive to NLs (Figure 4a,b). Furthermore, these sensitive modules specific to health status included different sets of endophytic bacteria and fungi. Alphaproteobacteria were the major endophytic bacterial class in modules 1 and 2, module 4 was dominated by Alphaproteobacteria and Planctomycetes, while module 5 contained only Bacilli and Myxococcia (Figure 4c). Dothideomycetes and Eurotiomycetes accounted for a large proportion of the endophytic fungi in modules 1, 2, and 4, and Taphrinomycetes and Dothideomycetes were highly abundant in module 5 (Figure 4d).

**Figure 5:**
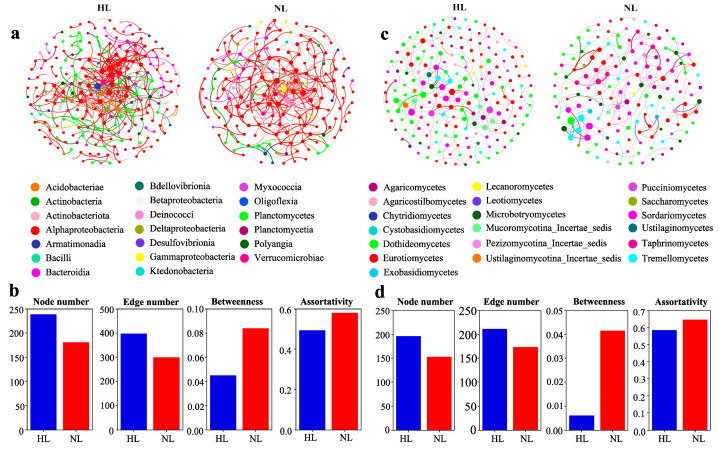
Co-occurrence network of endophytic bacterial (**a**) and fungal (**c**) communities in healthy leaves (HLs) and non-healthy leaves (NLs). A connection indicates strong (Spearman’s r > 0.8 for bacteria and r > 0.7 for fungi) and significant (*p* < 0.01) correlations. The nodes of each network are colored by class, and the size of each node indicates the connection number (that is, the degree). The edges between the nodes indicate the correlations, and the thickness of the edges is proportional to the weight of each correlation. Also shown are the numbers of nodes and edges and the degree of betweenness and assortativity of endophytic bacteria (**b**) and fungi (**d**) in the HL and NL co-occurrence patterns.

The networks of endophytic bacterial and fungal communities in leaves of different health status demonstrated distinct co-occurrence patterns (Figure 5a,c). The number of nodes and edges and the degree of betweenness and assortativity were used as network topological parameters to assess the complexity of the endophytic bacterial and fungal networks, with higher node and edge numbers and lower betweenness and assortativity representing greater network complexity. The results showed that poor leaf health status reduced the complexity of the endophytic bacterial and fungal community networks (Figure 5b,d).

### 3.4. Assembly Process of the Leaf Endophytic Microbial Community

**Figure 6:**
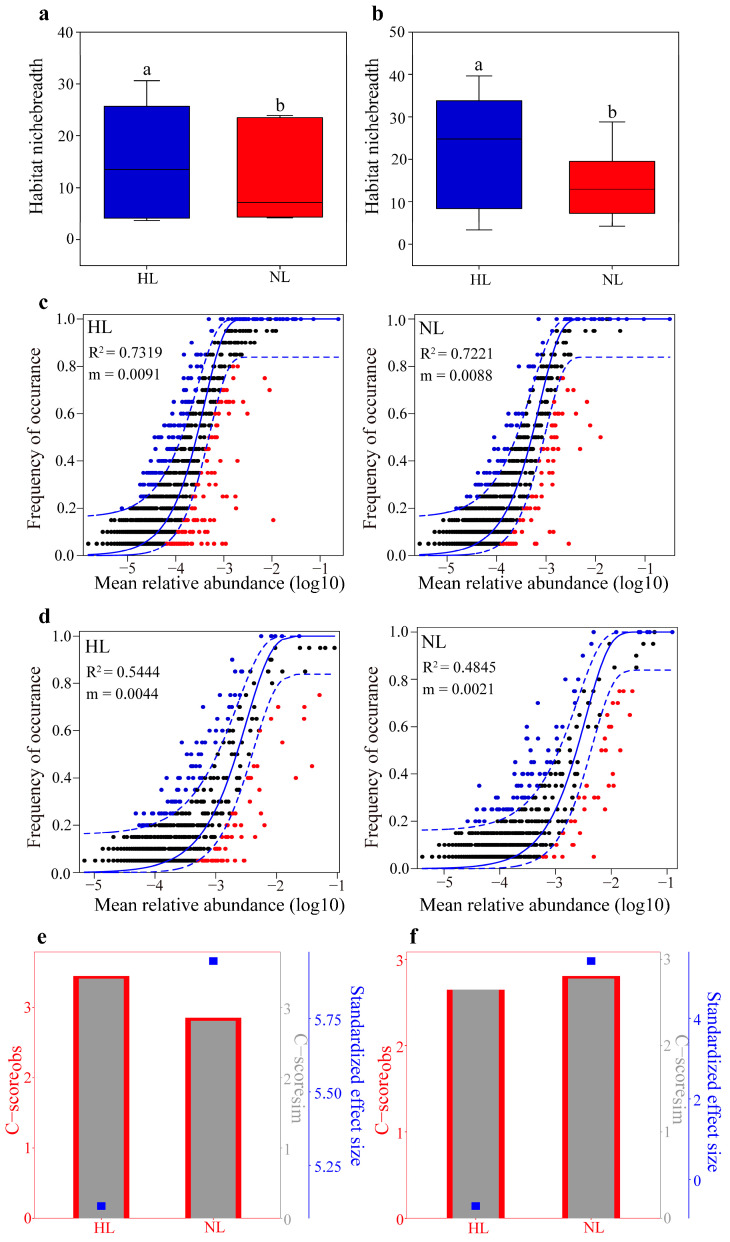
Ecological processes shaping the assembly of endophytic microbial communities in healthy leaves (HLs) and non-healthy leaves (NLs). The habitat niche breadths of endophytic bacteria (**a**) and fungi (**b**) in HLs and NLs. Different letters indicate significant differences at the *p* < 0.05 level according to the Wilcoxon rank-sum test. The neutral community model was used to assess the predicted occurrence frequencies of endophytic bacterial (**c**) and fungal (**d**) ASVs in HLs and NLs. ASVs that occur more frequently than predicted by the model are shown as blue dots, ASVs that occur less frequently than predicted are shown as red dots, and ASVs that occur within the predicted ranges are shown as black dots. The solid blue lines indicate the best fit of the model predictions, and the dashed blue lines represent 95% confidence intervals. R^2^ indicates the goodness of fit of the model, and the m value indicates the migration rate. C-scores of endophytic bacteria (**e**) and fungi (**f**) calculated using null models. Observed C-scores (C-score_obs_) that are higher than the simulated C-score (C-score_sim)_ indicate nonrandom co-occurrence patterns, and standardized effect size (SES) < −2 and >2 represent aggregation and segregation, respectively.

Estimates of habitat niche breadth showed that significantly higher mean values were observed in the endophytic bacterial and fungal communities of HLs than in those of NLs (Figure 6a,b). The endophytic bacterial and endophytic fungal communities from HLs (bacteria, R^2^ = 0.7319; fungi, R^2^ = 0.5444; Figure 6c) fit the neutral modes better than those from NLs (bacteria, R^2^ = 0.7221; fungi, R^2^ = 0.4845; Figure 6d), suggesting that the contribution of the endophytic bacterial and fungal communities was more strongly influenced by deterministic processes in the NLs. The C-score results showed that the value of standardized effect size (SES) of the endophytic bacterial and fungal communities was greater in NLs than in HLs (Figure 6e,f), which also indicated that the deterministic processes became more important in shaping the endophytic bacterial and fungal communities as leaf health decreased in *A. fanjingshanensis*. In addition, NCM indicated that a greater proportion of the relationship between the occurrence frequency of ASVs and their relative abundance was in the endophytic bacterial community than in the endophytic fungal community (Figure S3).

## 4. Discussion

The present study showed that the alpha diversity of the endophytic microbial community was significantly greater in healthy leaves than in non-healthy leaves, and these results corroborate previous studies showing that a greater proportion of the endophytic microbiome is found in healthy plant tissues [56,57]. Healthy plant tissues often provide a stable nutrient supply for the recruitment and establishment of the plant endophytic microbiome and present a complex and challenging environment for pathogen entry; in contrast, studies have suggested that plant tissues are more susceptible to colonization by some pathotrophic and saprotrophic microbial species after the vitality of plant tissues deteriorates or when a plant dies [56,58]; these species subsequently rapidly occupy the limited ecological niches available within the plant tissues, reducing the colonization of other endophytic microbiomes. Furthermore, the results of NMDS and PERMANOVA showed that the leaf endophytic microbial community significantly differed between healthy and non-healthy leaves, which is consistent with the findings of previous studies in the leaves of *Olea europaea* [19] and Gala apple [59]. Possible explanations for the changes in leaf endophytic microbial communities may be related to the differences in leaf physical characteristics, chemical defense mechanisms, and nutrient contents between healthy and non-healthy leaves [19,36,37,38]. In addition, certain leaf endophytic bacteria or fungi may switch from a commensalistic or mutualistic lifestyle to a pathogenic lifestyle when host leaves are in poor health [60], leading to the possible incorporation of new microbial species and to changes in the leaf endophytic microbial community.

Microbial interactions are important predictors of the mechanisms by which microorganisms respond to plant health status [17,61]. Co-occurrence network analysis not only can be used to describe the potentially complex interactions among microbial members but is also a useful tool for exploring the microbial co-occurrence patterns and highlighting the role of sensitive members [46,62]. In the meta co-occurrence networks of leaf endophytic microbial communities, we found that many hsASVs grouped into four distinct modules, and leaves with different health statuses were differentially enriched in these major modules. Specifically, numerous hsASVs assigned to healthy leaves were mainly located in modules 1, 2, and 4, whereas module 5 primarily contained hsASVs associated with non-healthy leaves. In addition, leaf decline tended to decrease the cumulative relative abundance of modules 1, 2, and 4 but increased the cumulative relative abundance of module 5. These results also confirmed that the distribution of endophytic ecological clusters could be altered by changes in leaf health status, which was documented in a previous study in which plant health status was shown to be important for the cumulative relative abundance of ecological clusters [21]. The indicator endophytic microbial taxa that responded to specific leaf health status were classified at the class level. For the endophytic bacterial community, Alphaproteobacteria were observed in higher abundance in healthy leaves. The Alphaproteobacteria class consists of many species that can form associations with plants and has been found in a wide range of environments, including soil, root nodules, and other plant tissues [63,64]. Coniferous species usually grow in nutrient-poor environments or where low temperatures limit soil N turnover, whereas leaf endophytes represent a low-cost, evolutionarily stable N^2^-fixing strategy that may enable conifers to cope with N limitation [65]. It is particularly intriguing that many alphaproteobacterial species have the ability to fix atmospheric N when associated with plants [65] and occur at high relative abundance in the endophytic bacterial communities of plant leaves [63], which may lead Alphaproteobacteria to be more abundant in healthy leaves. However, the non-healthy leaves contained only Bacilli and Myxococcia. Bacilli are likely to be involved in the induction of plant defense responses by suppressing plant endophytic pathogens when the plant is in poor health [66], and some members of Myxococcia can achieve competitive exclusion by producing antimicrobials [67]. For the endophytic fungal community, Dothideomycetes and Eurotiomycetes were found to be the major classes of fungi in plant leaves [68]. In addition, Dothideomycetes have also been found in decaying leaves [69], and a previous study showed that Dothideomycetes from living leaves grew faster than those isolated from non-living leaves [70], resulting in a greater proportion of Dothideomycetes in the healthy leaves. Taphrinomycetes were more abundant in non-healthy leaves, possibly because members of Taphrinomycetes are generally recognized as phytopathogens in higher plants [71].

The complexity of endophytic bacterial and fungal networks in leaves of different health status was further explored. Generally, network topological parameters, including the number of nodes and edges and the degree of betweenness and assortativity, have been used to assess the complexity of microbial communities [62]. In this study, we found that healthy leaves had a greater number of nodes and edges and lower betweenness and assortativity than non-healthy leaves, indicating that leaf decline can reduce the complexity of endophytic bacterial and fungal communities. This result is consistent with findings showing that the microbial community of healthy tomatoes formed a larger and more complex network than that of *Fusarium* wilt diseased tomatoes [57]. One explanation is that high microbial alpha diversity improves network complexity [62], and non-healthy leaves have lower endophytic microbial diversity than healthy leaves, which may contribute to the decrease in network complexity. Another explanation is that the carbohydrates produced by plants are important nutrient sources for the growth and colonization of endophytic microorganisms [7,72], but once leaves have declined, the microbial nutrient supply may depend mainly on the decomposition of dying leaves; however, this process often requires a long time, and reduced resource availability usually simplifies the microbial network complexity [73]. Furthermore, our study showed that the network of leaf endophytic bacteria was more complex than that of endophytic fungi in both healthy and non-healthy leaves. Bacteria have smaller cell sizes, greater local species richness, and faster growth rates than fungi [74,75], which may allow bacteria that originate from rainwater and bioaerosols from dust and other particles in the atmosphere to enter the leaf tissues through stomata more easily and establish more successfully than fungi, resulting in a more complex network.

The relative importance of stochastic and deterministic processes in governing leaf endophytic microbial community assembly shifted with leaf health status in *A. fanjingshanensis*, and the results may be critical to attain a mechanistic understanding of biodiversity conservation and community stability. NCM and the null model are two valid approaches that have been successfully applied to infer ecological processes acting on microbial community assembly [25,76]. Our NCM analysis demonstrated that the contribution of stochastic processes to leaf endophytic bacterial community assembly was greater than that to leaf endophytic fungal community assembly. Numerous previous studies have also pointed out that stochastic processes play a prominent role in shaping bacterial community assembly and that the fungal community is governed by deterministic processes in both soil and roots [25,27,28]. A reason for these observations could be that smaller microbes (e.g., bacteria) with higher dispersal rates are largely influenced by stochastic processes, whereas larger organisms (e.g., fungi) with more limited dispersal ability increase the importance of environmental filtering in determining microbial community assembly [74]. More importantly, the NCM and null model results showed that stochastic processes became slightly less important in shaping the leaf endophytic microbial community as leaf decline occurs in *A. fanjingshanensis*. There are three possible explanations for this trend. First, healthy leaves provide a more favorable environment for the growth and reproduction of endophytic microbes, resulting in a greater relative contribution of stochastic processes to endophytic microbial community assembly in healthy leaves. However, leaf decline not only alters the leaf microhabitat [19,38] but also increases the possibility of interspecies competition due to the colonization and unbalanced proliferation of plant pathogens and certain decomposing microorganisms [2,13], which may enhance the role of deterministic processes in endophytic microbial community assembly in non-healthy leaves. Second, the endophytic microbial communities in healthy leaves had a significantly greater niche breadth than those in non-healthy leaves based on the results of community-level habitat niche breadth, whereas a habitat generalist with a broader niche might be less influenced by deterministic processes [77]. Third, most leaf-colonizing endophytic microbes are more likely to be horizontally transmitted, with the main routes being from the plant roots and from the phyllosphere [78,79]. A high transpiration stream and stomatal number may increase the possibility of the movement and successful establishment of endophytic microbes [80,81], which is why we observed slightly higher m values in healthy leaves than in non-healthy leaves, and this finding is supported by the previous studies showing that a high dispersal rate may enhance the relative importance of stochastic processes [27,82]. However, in this study, we collected samples of leaves with only two health statuses, and further research on the complete process by which leaves progress from healthy to dead is needed to explore the balance between stochastic and deterministic processes in endophytic microbial communities as leaves decline.

## 5. Conclusions

The leaf endophytic microbial communities of *A. fanjingshanensis* showed considerable responses to declines in leaf health status, as illustrated by decreased leaf endophytic bacterial and fungal diversity, altered community composition and structure, reduced habitat niche breadth, influenced the relative importance of stochastic and deterministic processes in determining the assembly of endophytic bacterial and fungal communities, altered species and abundance of leaf endophytic microbial hsASVs, and the reduced complexity of the endophytic bacterial and fungal networks. These findings increase our understanding of the interaction between endophytic microbes and changes in leaf health status and provide the potential for manipulating specific microbiomes (e.g., well-known beneficial endophytic microbial taxa, hsASVs, and even highly connected taxa located in the endophytic microbial network) in follow-up experiments to promote plant health and realize the conservation of endangered plant species.

## Data Availability

The raw sequencing data have been deposited in the National Center for Biotechnology Information Sequence Read Archive under the accession number PRJNA1121495. ASV tables for the leaf endophytic microbial community are available from the corresponding author upon reasonable request.

## Supplementary Materials

The following supporting information can be downloaded at: https://www.mdpi.com/article/10.3390/microorganisms12071254/s1, Figure S1: Rarefaction curves showing the relationship between the number of endophytic bacterial (a) and fungal (b) ASVs and the number of sequences from all 40 leaf samples; Figure S2: Defining network modules; Figure S3: The neutral community model was used to assess the predicted occurrence frequencies of endophytic bacterial (a) and fungal (b) ASVs in the leaves of *A. fanjingshanensis*; Table S1: The indicator species and edgeR results and the assignments to health-sensitive ASVs of leaf endophytic bacteria; Table S2: The indicator species and edgeR results and the assignments to health-sensitive ASVs of leaf endophytic fungi; Table S3: Properties of leaf meta co-occurrence networks.
