# Supplementary material for: Leaf Health Status Regulates Endophytic Microbial Community Structure, Network Complexity, and Assembly Processes in the Leaves of the Rare and Endangered Plant Species Abies fanjingshanensis
Source: Microorganisms. 2024 Jun 21;12(7):1254. doi: 10.3390/microorganisms12071254 (PMC11279022; doi:10.3390/microorganisms12071254)
Supplement: Supplementary file 1 [file microorganisms-12-01254-s001.zip › Supporting information .pdf]

Figures

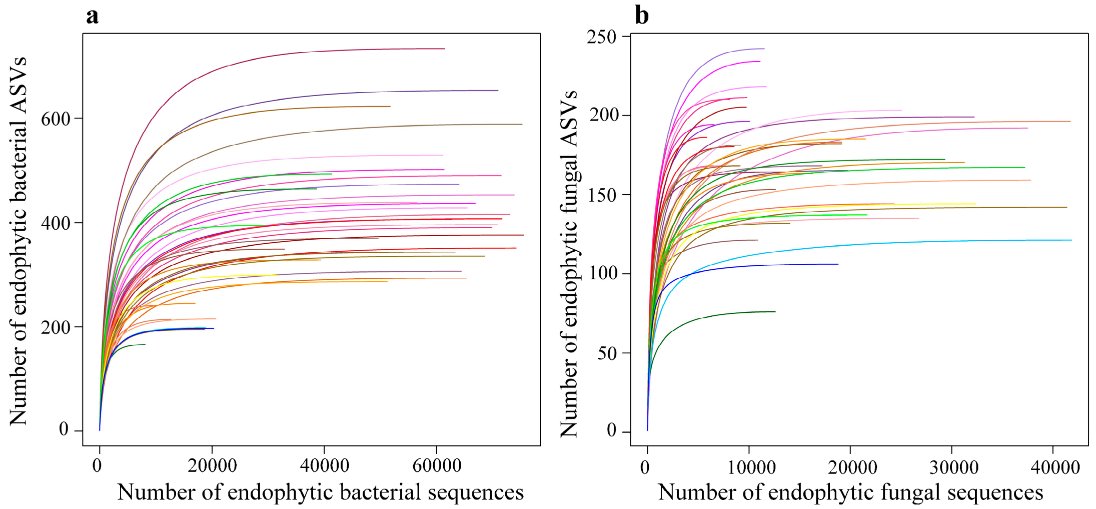

Figure S1. Rarefaction curves showing the relationship between the number of endophytic bacterial (**a**) and fungal (**b**) ASVs and the number of sequences from all 40 leaf samples. Each curve represents rarefaction of a single leaf sample.

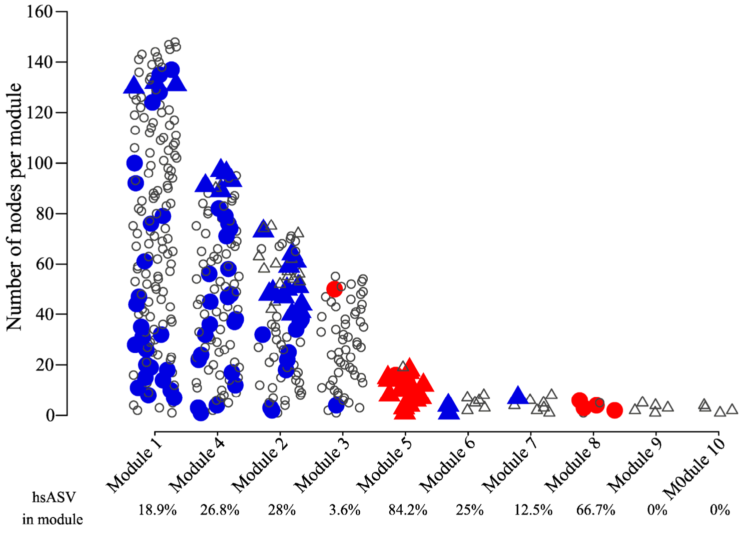

Figure S2. Defining network modules. Plots showing the number of ASVs in the top 10 most populated modules for the leaf meta co-occurrence networks. Circles and triangles represent endophytic bacterial and fungal ASVs, respectively. The sensitive endophytic bacterial and fungal ASVs that are abundant under a specific leaf health status are coloured by blue for the healthy leaves and red for the non-healthy leaves, and the gray open symbols represent the endophytic bacterial and fungal ASVs that are insensitive to leaf health status.

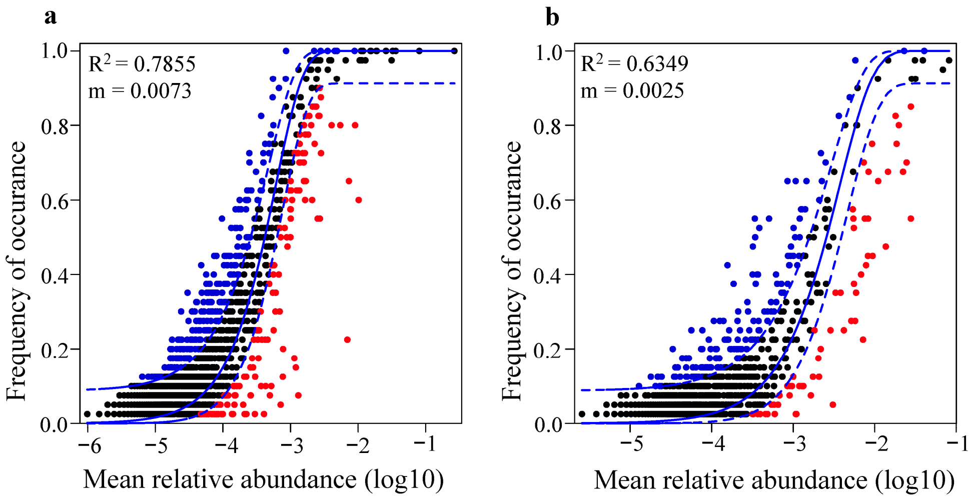

Figure S3. The neutral community model was used to assess the predicted occurrence frequencies of endophytic bacterial (**a**) and fungal (**b**) ASVs in the leaves of *A. fanjingshanensis*. ASVs that occur more frequently than predicted by the model are shown as blue dots, ASVs that occur less frequently than predicted are shown as red dots, and ASVs that occur within the predicted ranges are shown as black dots. The solid blue lines indicate the best fit of the model predictions, and the dashed blue lines represent 95% confidence intervals. R^2 indicates the goodness of fit of the model, and the m value indicates the migration rate.

Tables

Table S1. The indicator species and edgeR results and the assignments to health-sensitive ASVs of leaf endophytic bacteria.

ASV_ID	Phylum	Class	Order	Family	Genus	HLs	NLs	Stat	*P*. value	edgeR	hsASV
ASV_13	Proteobacteria	Alphaproteobacteria	Rhizobiales	Beijerinckiaceae	1174-901-12	0	1	0.5496	0.0001	1	1
ASV_25	Proteobacteria	Betaproteobacteria	Betaproteobacteria_ unclassified	Betaproteobacteria_ unclassified	Betaproteobacteria_ unclassified	1	0	0.3999	0.0112	1	1
ASV_30	Proteobacteria	Alphaproteobacteria	Caulobacterales	Caulobacteraceae	*Caulobacter*	1	0	0.3221	0.0422	1	1
ASV_32	Bacteroidota	Bacteroidia	Cytophagales	Hymenobacteraceae	*Hymenobacter*	1	0	0.3546	0.0249	1	1
ASV_34	Deinococcota	Deinococci	Deinococcales	Deinococcaceae	*Deinococcus*	1	0	0.4359	0.0043	1	1
ASV_35	Bacteroidota	Bacteroidia	Cytophagales	Hymenobacteraceae	*Hymenobacter*	1	0	0.4124	0.0088	1	1
ASV_39	Deinococcota	Deinococci	Deinococcales	Deinococcaceae	*Deinococcus*	1	0	0.3466	0.0303	1	1
ASV_40	Proteobacteria	Alphaproteobacteria	Sphingomonadales	Sphingomonadaceae	*Polymorphobacter*	1	0	0.4550	0.0027	1	1
ASV_41	Proteobacteria	Alphaproteobacteria	Rhizobiales	Beijerinckiaceae	1174-901-12	1	0	0.4405	0.0043	1	1
ASV_57	Proteobacteria	Alphaproteobacteria	Acetobacterales	Acetobacteraceae	Acetobacteraceae_ unclassified	1	0	0.4435	0.0046	1	1
ASV_60	Proteobacteria	Alphaproteobacteria	Rhodospirillales	Rhodospirillales_ unclassified	Rhodospirillales_ unclassified	1	0	0.3716	0.0148	1	1
ASV_62	Proteobacteria	Alphaproteobacteria	Sphingomonadales	Sphingomonadaceae	Sphingomonadaceae _unclassified	1	0	0.5454	0.0004	1	1
ASV_66	Bacteroidota	Bacteroidia	Cytophagales	Hymenobacteraceae	*Hymenobacter*	1	0	0.5638	0.0002	1	1
ASV_67	Proteobacteria	Betaproteobacteria	Betaproteobacteria_ unclassified	Betaproteobacteria_ unclassified	Betaproteobacteria_ unclassified	1	0	0.4789	0.0024	1	1
ASV_69	Proteobacteria	Alphaproteobacteria	Sphingomonadales	Sphingomonadaceae	*Sphingomonas*	1	0	0.5744	0.0001	1	1
ASV_71	Bacteroidota	Bacteroidia	Cytophagales	Hymenobacteraceae	*Hymenobacter*	1	0	0.4829	0.0019	1	1

ASV_82	Bacteroidota	Bacteroidia	Cytophagales	Hymenobacteraceae	*Hymenobacter*	1	0	0.4182	0.0072	1	1
ASV_89	Proteobacteria	Alphaproteobacteria	Sphingomonadales	Sphingomonadaceae	*Sphingomonas*	1	0	0.4649	0.0037	1	1
ASV_99	Bacteroidota	Bacteroidia	Cytophagales	Hymenobacteraceae	*Hymenobacter*	1	0	0.5229	0.0005	1	1
ASV_100	Bacteroidota	Bacteroidia	Cytophagales	Hymenobacteraceae	*Hymenobacter*	1	0	0.5747	0.0005	1	1
ASV_109	Bacteroidota	Bacteroidia	Cytophagales	Hymenobacteraceae	*Hymenobacter*	1	0	0.5900	0.0001	1	1
ASV_125	Proteobacteria	Alphaproteobacteria	Acetobacterales	Acetobacteraceae	*Acidiphilium*	1	0	0.3926	0.0131	1	1
ASV_129	Bacteroidota	Bacteroidia	Cytophagales	Hymenobacteraceae	*Hymenobacter*	1	0	0.4534	0.0037	1	1
ASV_136	Bacteroidota	Bacteroidia	Cytophagales	Hymenobacteraceae	*Hymenobacter*	1	0	0.4619	0.0029	1	1
ASV_163	Bacteroidota	Bacteroidia	Cytophagales	Hymenobacteraceae	*Hymenobacter*	1	0	0.3205	0.0449	1	1
ASV_166	Proteobacteria	Alphaproteobacteria	Rhodospirillales	Rhodospirillales_ unclassified	Rhodospirillales_ unclassified	1	0	0.4591	0.0043	1	1
ASV_167	Firmicutes	Bacilli	Bacillales	Bacillaceae	*Bacillus*	0	1	0.5265	0.0014	1	1
ASV_168	Myxococcota	Polyangia	Haliangiales	Haliangiaceae	*Haliangium*	1	0	0.3787	0.0167	1	1
ASV_183	Bacteroidota	Bacteroidia	Cytophagales	Hymenobacteraceae	*Hymenobacter*	1	0	0.3522	0.0228	1	1
ASV_189	Verrucomicrobiota	Verrucomicrobiae	Chthoniobacterales	Chthoniobacteraceae	LD29	1	0	0.4606	0.0040	1	1
ASV_196	Proteobacteria	Alphaproteobacteria	Rhodospirillales	Acetobacteraceae	Acetobacteraceae_ unclassified	1	0	0.4796	0.0022	1	1
ASV_234	Planctomycetota	Planctomycetes	Isosphaerales	Isosphaeraceae	*Tundrisphaera*	1	0	0.4006	0.0108	1	1
ASV_249	Bacteroidota	Bacteroidia	Cytophagales	Hymenobacteraceae	*Hymenobacter*	1	0	0.4589	0.0021	1	1
ASV_255	Proteobacteria	Alphaproteobacteria	Acetobacterales	Acetobacteraceae	*Acidiphilium*	1	0	0.4350	0.0053	1	1
ASV_268	Planctomycetota	Planctomycetia	Planctomycetales	Isosphaeraceae	*Singulisphaera*	1	0	0.3699	0.0188	1	1
ASV_278	Planctomycetota	Planctomycetes	Isosphaerales	Isosphaeraceae	*Tundrisphaera*	1	0	0.3860	0.0107	1	1
ASV_280	Myxococcota	Polyangia	Polyangiales	Phaselicystidaceae	*Phaselicystis*	1	0	0.5221	0.0004	1	1
ASV_294	Proteobacteria	Alphaproteobacteria	Rhizobiales	Beijerinckiaceae	1174-901-12	1	0	0.4391	0.0037	1	1
ASV_306	Bdellovibrionota	Bdellovibrionia	Bdellovibrionales	Bdellovibrionaceae	*Bdellovibrio*	1	0	0.4765	0.0017	1	1
ASV_311	Bacteroidota	Bacteroidia	Cytophagales	Hymenobacteraceae	*Hymenobacter*	1	0	0.4673	0.0014	1	1

ASV_312	Proteobacteria	Deltaproteobacteria	Myxococcales	Archangiaceae	Archangiaceae_ unclassified	1	0	0.4019	0.0093	1	1
ASV_317	Planctomycetota	Planctomycetes	Isosphaerales	Isosphaeraceae	*Tundrisphaera*	1	0	0.4084	0.0072	1	1
ASV_334	Deinococcota	Deinococci	Deinococcales	Deinococcaceae	*Deinococcus*	1	0	0.3892	0.0124	1	1
ASV_343	Proteobacteria	Alphaproteobacteria	Sphingomonadales	Sphingomonadaceae	*Polymorphobacter*	1	0	0.3404	0.0332	1	1
ASV_356	Planctomycetota	Planctomycetes	Isosphaerales	Isosphaeraceae	*Tundrisphaera*	1	0	0.4346	0.0055	1	1
ASV_361	Acidobacteriota	Acidobacteriae	Acidobacteriales	Acidobacteriaceae_ (Subgroup_1)	*Terriglobus*	1	0	0.3600	0.0273	1	1
ASV_364	Proteobacteria	Gammaproteobacteria	Pseudomonadales	Pseudomonadaceae	*Pseudomonas*	0	1	0.5008	0.0020	1	1
ASV_369	Myxococcota	Myxococcia	Myxococcales	Myxococcaceae	P3OB-42	0	1	0.5346	0.0002	1	1
ASV_374	Proteobacteria	Alphaproteobacteria	Acetobacterales	Acetobacteraceae	*Rhodovastum*	1	0	0.3583	0.0250	1	1
ASV_381	Proteobacteria	Alphaproteobacteria	Rhodobacterales	Rhodobacteraceae	*Amylibacter*	0	1	0.4749	0.0014	1	1
ASV_398	Myxococcota	Polyangia	Haliangiales	Haliangiaceae	*Haliangium*	1	0	0.3246	0.0407	1	1
ASV_447	Planctomycetota	Planctomycetes	Isosphaerales	Isosphaeraceae	*Tundrisphaera*	1	0	0.3448	0.0278	1	1
ASV_475	Planctomycetota	Planctomycetia	Planctomycetales	Isosphaeraceae	*Singulisphaera*	1	0	0.3443	0.0309	1	1
ASV_487	Verrucomicrobiota	Verrucomicrobiae	Chthoniobacterales	Chthoniobacteraceae	LD29	1	0	0.5785	0.0003	1	1
ASV_491	Firmicutes	Bacilli	Lactobacillales	Enterococcaceae	*Enterococcus*	0	1	0.4977	0.0013	1	1
ASV_528	Proteobacteria	Alphaproteobacteria	Acetobacterales	Acetobacteraceae	*Acidiphilium*	1	0	0.4537	0.0029	1	1
ASV_558	Acidobacteriota	Acidobacteriota_ unclassified	Acidobacteriota_ unclassified	Acidobacteriota_ unclassified	Acidobacteriota_ unclassified	1	0	0.3375	0.0310	1	1
ASV_566	Proteobacteria	Oligoflexia	Silvanigrellales	Silvanigrellales_ unclassified	Silvanigrellales_ unclassified	1	0	0.4758	0.0012	1	1
ASV_582	Bacteroidota	Bacteroidia	Sphingobacteriales	Sphingobacteriaceae	*Mucilaginibacter*	1	0	0.5051	0.0010	1	1
ASV_603	Proteobacteria	Gammaproteobacteria	Legionellales	Legionellaceae	*Legionella*	1	0	0.4031	0.0088	1	1
ASV_637	Myxococcota	Myxococcia	Myxococcales	Myxococcaceae	P3OB-42	1	0	0.3781	0.0173	1	1
ASV_666	Firmicutes	Bacilli	Lactobacillales	Lactobacillaceae	*Ligilactobacillus*	0	1	0.4264	0.0087	1	1

ASV_701	Bacteroidota	Bacteroidia	Cytophagales	Hymenobacteraceae	*Hymenobacter*	1	0	0.3669	0.0177	1	1
ASV_719	Bacteroidota	Bacteroidia	Cytophagales	Spirosomaceae	*Huanghella*	1	0	0.4280	0.0060	1	1

Table S2. The indicator species and edgeR results and the assignments to health-sensitive ASVs of leaf endophytic fungi.

ASV_ID	Phylum	Class	Order	Family	Genus	HLs	NLs	Stat	P.value	edgeR	hsASV
ASV_8	Ascomycota	Dothideomycetes	Botryosphaeriales	Botryosphaeriaceae	*Microdiplodia*	1	0	0.5136	0.0007	1	1
ASV_12	Ascomycota	Dothideomycetes	Capnodiales	Capnodiales_ unclassified	Capnodiales_ unclassified	1	0	0.6286	0.0001	1	1
ASV_13	Ascomycota	Dothideomycetes	Capnodiales	Capnodiales_ unclassified	Capnodiales_ unclassified	1	0	0.6151	0.0001	1	1
ASV1_115	Ascomycota	Dothideomycetes	Dothideomycetes_ Incertae_sedis	Dothideomycetes_ Incertae_sedis	*Peltaster*	1	0	0.4089	0.0091	1	1
ASV_116	Ascomycota	Dothideomycetes	Dothideomycetes_ Incertae_sedis	Dothideomycetes_ Incertae_sedis	*Peltaster*	0	1	0.4478	0.0039	1	1
ASV_122	Ascomycota	Dothideomycetes	Dothideomycetes_ unclassified	Dothideomycetes_ unclassified	Dothideomycetes_ unclassified	0	1	0.7318	0.0001	1	1
ASV_124	Ascomycota	Dothideomycetes	Dothideomycetes_ unclassified	Dothideomycetes_ unclassified	Dothideomycetes_ unclassified	0	1	0.4565	0.0044	1	1
ASV_128	Ascomycota	Dothideomycetes	Dothideomycetes_ unclassified	Dothideomycetes_ unclassified	Dothideomycetes_ unclassified	0	1	0.3648	0.0227	1	1
ASV_129	Ascomycota	Dothideomycetes	Dothideomycetes_ unclassified	Dothideomycetes_ unclassified	Dothideomycetes_ unclassified	1	0	0.4972	0.0019	1	1
ASV_270	Ascomycota	Dothideomycetes	Myriangiales	Elsinoaceae	*Elsinoe*	0	1	0.4750	0.0005	1	1
ASV_344	Ascomycota	Dothideomycetes	Pleosporales	Pleosporales_	Pleosporales_	1	0	0.5047	0.0005	1	1

				unclassified	unclassified						
ASV_347	Ascomycota	Dothideomycetes	Pleosporales	Pleosporales_ unclassified	Pleosporales_ unclassified	1	0	0.5134	0.0007	1	1
ASV_417	Ascomycota	Eurotiomycetes	Chaetothyriales	Chaetothyriales_ Incertae_sedis	*Strelitziana*	1	0	0.4033	0.0122	1	1
ASV_443	Ascomycota	Eurotiomycetes	Chaetothyriales	Chaetothyriales_ unclassified	Chaetothyriales_ unclassified	1	0	0.4520	0.0042	1	1
ASV_465	Ascomycota	Eurotiomycetes	Chaetothyriales	Herpotrichiellaceae	*Cladophialophora*	1	0	0.4716	0.0023	1	1
ASV_466	Ascomycota	Eurotiomycetes	Chaetothyriales	Herpotrichiellaceae	*Cladophialophora*	1	0	0.5556	0.0005	1	1
ASV_469	Ascomycota	Eurotiomycetes	Chaetothyriales	Herpotrichiellaceae	*Cladophialophora*	1	0	0.5607	0.0003	1	1
ASV_519	Ascomycota	Eurotiomycetes	Chaetothyriales	Herpotrichiellaceae	*Cladophialophora*	1	0	0.4857	0.0017	1	1
ASV_520	Ascomycota	Eurotiomycetes	Chaetothyriales	Herpotrichiellaceae	*Cladophialophora*	1	0	0.3875	0.0145	1	1
ASV_573	Ascomycota	Eurotiomycetes	Chaetothyriales	Herpotrichiellaceae	*Cladophialophora*	1	0	0.3276	0.0370	1	1
ASV_630	Ascomycota	Eurotiomycetes	Chaetothyriales	Herpotrichiellaceae	Herpotrichiellaceae_ unclassified	1	0	0.3672	0.0224	1	1
ASV_1330	Ascomycota	Sordariomycetes	Xylariales	Xylariales_ unclassified	Xylariales_ unclassified	1	0	0.5186	0.0008	1	1
ASV_1346	Ascomycota	Taphrinomycetes	Taphrinales	Taphrinaceae	*Lalaria*	0	1	0.6594	0.0001	1	1
ASV_1347	Ascomycota	Taphrinomycetes	Taphrinales	Taphrinaceae	*Lalaria*	0	1	0.6518	0.0001	1	1
ASV_1348	Ascomycota	Taphrinomycetes	Taphrinales	Taphrinaceae	*Lalaria*	0	1	0.5510	0.0005	1	1
ASV_1357	Ascomycota	Taphrinomycetes	Taphrinales	Taphrinaceae	*Lalaria*	0	1	0.7050	0.0001	1	1
ASV_1358	Ascomycota	Taphrinomycetes	Taphrinales	Taphrinaceae	*Lalaria*	0	1	0.3615	0.0240	1	1
ASV_1359	Ascomycota	Taphrinomycetes	Taphrinales	Taphrinaceae	*Lalaria*	0	1	0.5462	0.0001	1	1
ASV_1360	Ascomycota	Taphrinomycetes	Taphrinales	Taphrinaceae	*Lalaria*	0	1	0.5001	0.0012	1	1
ASV_1375	Ascomycota	Taphrinomycetes	Taphrinales	Taphrinaceae	*Taphrina*	0	1	0.5203	0.0013	1	1
ASV_1407	Ascomycota	Taphrinomycetes	Taphrinales	Taphrinaceae	Taphrinaceae_	0	1	0.4930	0.0011	1	1

					unclassified						
ASV_1589	Basidiomycota	Agaricomycetes	Agaricomycetes_ unclassified	Agaricomycetes_ unclassified	Agaricomycetes_ unclassified	1	0	0.4680	0.0030	1	1
ASV_1594	Basidiomycota	Agaricomycetes	Agaricomycetes_ unclassified	Agaricomycetes_ unclassified	Agaricomycetes_ unclassified	1	0	0.3960	0.0136	1	1
ASV_1906	Basidiomycota	Agaricostilbomycetes	Agaricostilbales	Agaricostilbaceae	Bensingtonia	1	0	0.4129	0.0080	1	1
ASV_1968	Basidiomycota	Microbotryomycetes	Microbotryomycetes _unclassified	Microbotryomycetes _unclassified	Microbotryomycetes _unclassified	0	1	0.5719	0.0001	1	1
ASV_2211	Basidiomycota	Ustilaginomycetes	Ustilaginales	Ustilaginaceae	*Pseudozyma*	1	0	0.3201	0.0472	1	1
ASV_2235	Chytridiomycota	Chytridiomycetes	Spizellomycetales	Spizellomycetaceae	*Spizellomyces*	1	0	0.5823	0.0003	1	1
ASV_2268	Zygomycota	Mucoromycotina_ Incertae_sedis	Mucorales	Mucoraceae	*Mucor*	1	0	0.3253	0.0415	1	1
ASV_8	Ascomycota	Dothideomycetes	Botryosphaeriales	Botryosphaeriaceae	*Microdiplodia*	1	0	0.5136	0.0007	1	1

Table S3. Properties of leaf meta co-occurrence networks.

**ASVs**[a]		**Connections**[b]			**Connectivity**[c]	**hsASVs**[d]	
**Bacteria**	**Fungi**	**Bac-Bac**	**Fun-Fun**	**Bac-Fun**	**Network wide**	**Bacteria**	**Fungi**
358	87	2492	136	45	12.01	64	38

[a]Number of network nodes
[b]Number of network edges
[c]Mean number of connections per nodes
[d]Number of health sensitive ASVs present in the network
