# Supplementary figures and images for: Leaf Health Status Regulates Endophytic Microbial Community Structure, Network Complexity, and Assembly Processes in the Leaves of the Rare and Endangered Plant Species Abies fanjingshanensis
Source: Microorganisms. 2024 Jun 21;12(7):1254. doi: 10.3390/microorganisms12071254 (PMC11279022; doi:10.3390/microorganisms12071254)

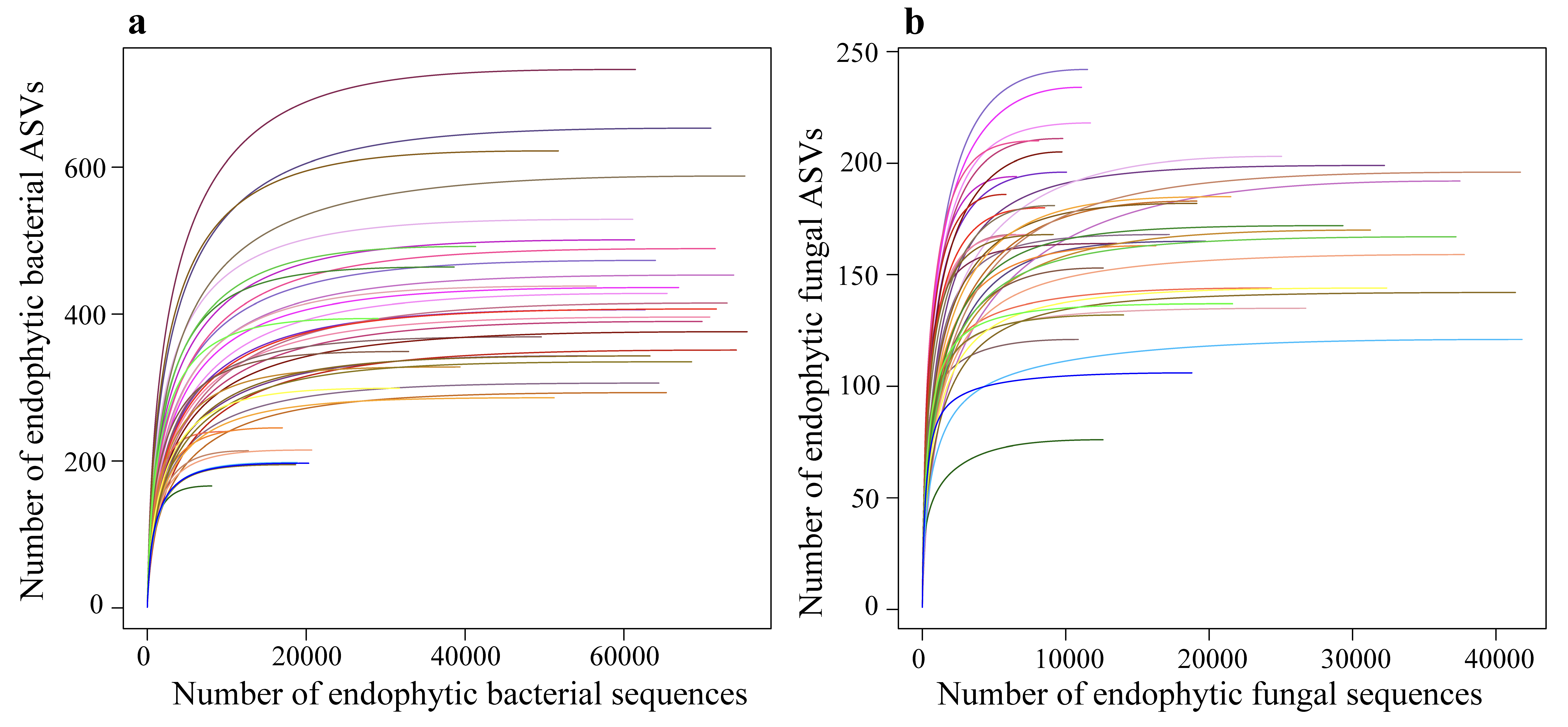

Supplement: Supplementary file 1 [file microorganisms-12-01254-s001.zip › FigureS1.tiff]

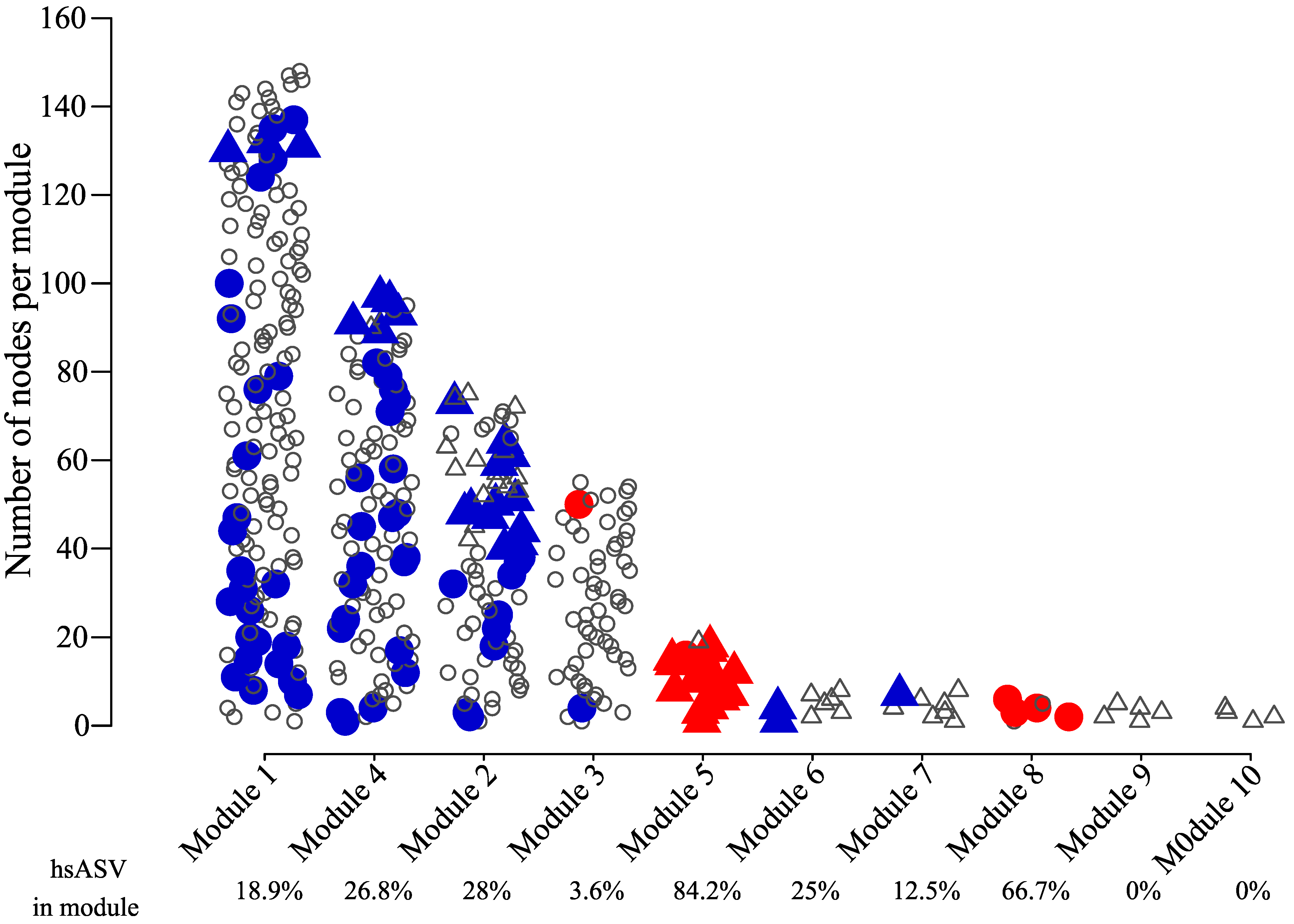

Supplement: Supplementary file 1 [file microorganisms-12-01254-s001.zip › FigureS2.tiff]

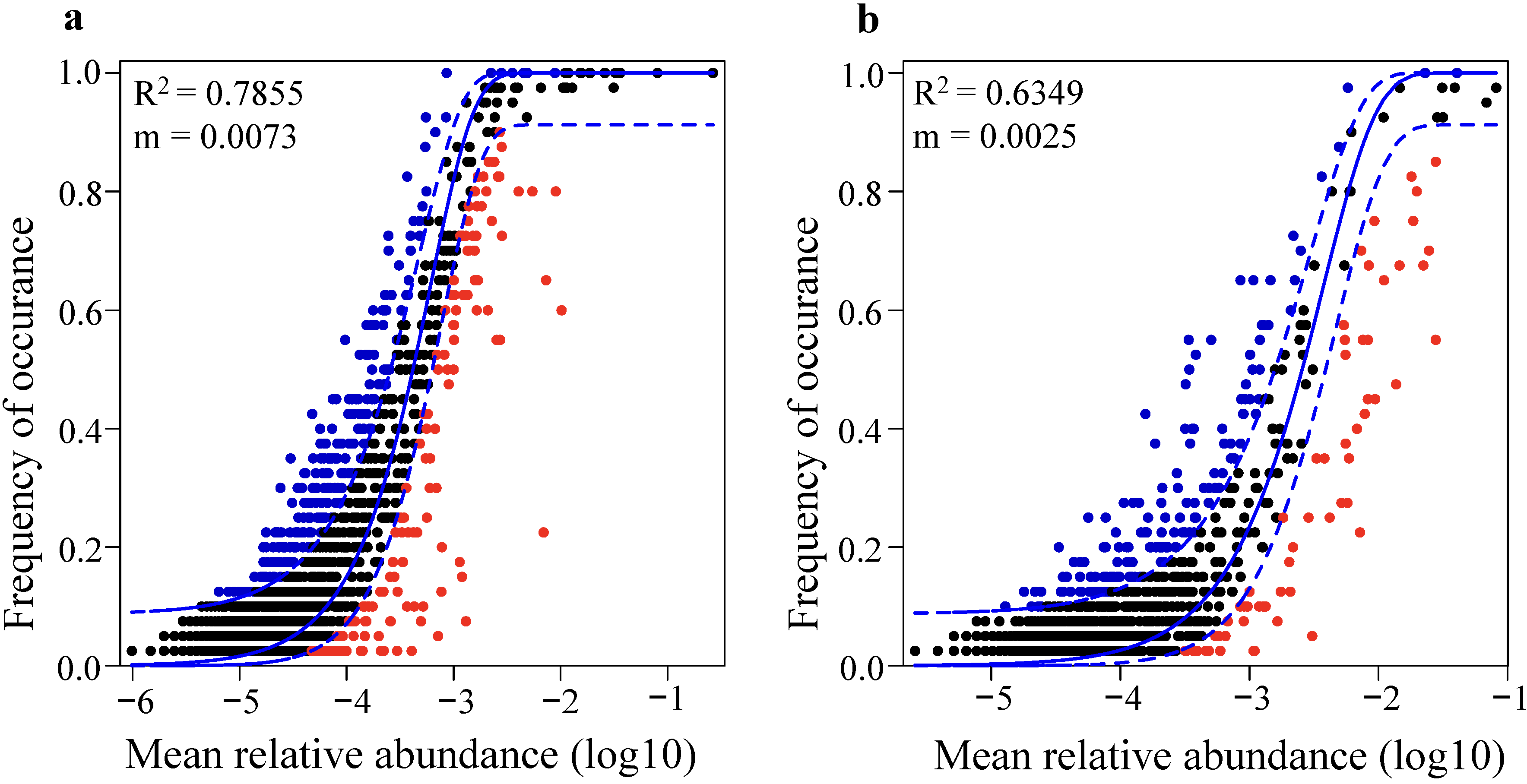

Supplement: Supplementary file 1 [file microorganisms-12-01254-s001.zip › FigureS3.tiff]
